# Brief inhalation of nitric oxide increases resuscitation success and improves 7-day-survival after cardiac arrest in rats: a randomized controlled animal study

**DOI:** 10.1186/s13054-015-1128-x

**Published:** 2015-11-17

**Authors:** Anne Brücken, Matthias Derwall, Christian Bleilevens, Christian Stoppe, Andreas Götzenich, Nadine T. Gaisa, Joachim Weis, Kay Wilhelm Nolte, Rolf Rossaint, Fumito Ichinose, Michael Fries

**Affiliations:** Department of Anesthesiology, University Hospital RWTH Aachen, Pauwelsstr. 30, 52074 Aachen, Germany; Department of Thoracic, Cardiac and Vascular Surgery, University Hospital RWTH Aachen, Pauwelsstr. 30, 52074 Aachen, Germany; Institute of Pathology, University Hospital RWTH Aachen, Pauwelsstr. 30, 52074 Aachen, Germany; Institute for Neuropathology, University Hospital RWTH Aachen, Pauwelsstr. 30, 52074 Aachen, Germany; Anesthesia Center for Critical Care Research, Department of Anesthesia, Critical Care, and Pain Medicine, Massachusetts General Hospital and Harvard Medical School, 55 Fruit Street, Boston, MA 02114 USA; Department of Anesthesiology, St. Vincenz Hospital Limburg, Auf dem Schafsberg, 65549 Limburg, Germany

## Abstract

**Introduction:**

Inhaled nitric oxide (iNO) improves outcomes when given post systemic ischemia/reperfusion injury. iNO given during cardiopulmonary resuscitation (CPR) may therefore improve return of spontaneous circulation (ROSC) rates and functional outcome after cardiac arrest (CA).

**Methods:**

Thirty male Sprague-Dawley rats were subjected to 10 minutes of CA and at least 3 minutes of CPR. Animals were randomized to receive either 0 (n = 10, Control), 20 (n = 10, 20 ppm), or 40 (n = 10, 40 ppm) ppm iNO during CPR until 30 minutes after ROSC. A neurological deficit score was assessed daily for seven days following the experiment. On day 7, brains, hearts, and blood were sampled for histological and biochemical evaluation.

**Results:**

During CPR, 20 ppm iNO significantly increased diastolic arterial pressure (Control: 57 ± 5.04 mmHg; 20 ppm: 71.57 ± 57.3 mmHg, p < 0.046) and decreased time to ROSC (Control: 842 ± 21 s; 20 ppm: 792 ± 5 s, (p = 0.02)).

Thirty minutes following ROSC, 20 ppm iNO resulted in an increase in mean arterial pressure (Control: 83 ± 4 mmHg; 20 ppm: 98 ± 4 mmHg, p = 0.035), a less pronounced rise in lactate and inflammatory cytokine levels, and attenuated cardiac damage. Inhalation of NO at 20 ppm improved neurological outcomes in rats 2 to 7 days after CA and CPR. This translated into increases in 7 day survival (Control: 4; 20 ppm: 10; 40 ppm 6, (p ≤ 0.05 20 ppm vs Control and 40 ppm).

**Conclusions:**

Our study revealed that breathing NO during CPR markedly improved resuscitation success, 7-day neurological outcomes and survival in a rat model of VF-induced cardiac arrest and CPR. These results support the beneficial effects of NO inhalation after cardiac arrest and CPR.

## Introduction

In industrialized countries, sudden cardiac arrest (CA) remains one of the leading causes of death [1]. Despite improvements in pre-hospital care and the introduction of mild therapeutic hypothermia (MTH) [2], mortality rates of out-of-hospital CA victims are still high, although regional variations are often reported [3, 4]. Survivors frequently suffer from moderate to severe cognitive deficits 3 months post resuscitation [5, 6]. These poor outcomes are mainly attributable to a distinct pathology termed post-CA syndrome [7]. Clinically, this syndrome becomes apparent as cerebral and myocardial dysfunction [8], often associated with a pronounced inflammatory response [9] in concert with microvascular alterations [10, 11] and adrenal dysfunction [9, 12].

The benefits of MTH have recently been questioned in a large study predominantly including patients with ventricular fibrillation and short duration ischemia [13]. Furthermore, in patients with a more severe insult presenting with asystole, MTH is probably even less effective [14]. Currently no pharmacological agent is available to further improve outcomes for CA victims.

Inhaled nitric oxide (iNO) is approved to treat newborn hypoxemia with pulmonary hypertension [15]. In addition, iNO has been used in the intensive care setting as a selective pulmonary vasodilator in patients suffering from acute respiratory distress syndrome and right heart failure [16, 17]. Accumulating evidence of preclinical and clinical studies suggests that iNO also exerts remote effects independent of its local vasodilatory action. Recently, Minamishima and colleagues demonstrated in a murine model that breathing 40 ppm NO starting 1 hour after CA and cardiopulmonary resuscitation (CPR) markedly improved neurological and myocardial function, and 10-day survival rate [18]. Whether or not administration of iNO during CPR improves resuscitation success rates was not addressed.

To accelerate the pace of translation of this promising treatment into the clinic to improve outcomes after CA, we examined the effects of iNO administered during CPR until 30 minutes after return of spontaneous circulation (ROSC). We hypothesized that iNO increases resuscitation success and improves cerebral and myocardial outcomes, and survival after prolonged CA in rats.

## Methods

The study protocol was approved by the appropriate ethical Institution (Landesamt für Natur, Umwelt und Verbraucherschutz Nordrhein-Westfalen; Recklinghausen; Germany). The experimental procedures were performed according to the Guide for the Care and Use of Laboratory Animals formulated by the National Research Council (National Academies Press, 1996) and the ARRIVE guidelines (National Centre for the Replacement, Refinement and Reduction of animals in research, 2010). In addition, all reported data and outcomes are in accordance with the Utstein style guidelines for uniform reporting of laboratory CPR research [19].

A total of 30 male Sprague-Dawley rats (Charles River, Sulzfeld, Germany) weighing between 400–500 g were investigated in an established rodent CA model [20]. Animals were housed in adequately spaced cages (60 cm × 40 cm; type 2000; Tecniplast; Buguggiate; Italy) with a 12-hour light-dark cycle. Animals had free access to water and food prior to the study.

Two different concentrations of iNO (20 ppm vs. 40 ppm), administered during CPR until 30 minutes after ROSC, were tested against 0 ppm iNO (control). Primary endpoints were resuscitation rates, and neurological outcome when compared to controls.

### Animal preparation

We used a rat model of CA and CPR as previously described, with minor modifications [20]. On the day of the procedure, rats were anesthetized with an intraperitoneal injection of pentobarbital (45 mg · kg^−1^). Additional doses (10 mg · kg^−1^) of pentobarbital were administered if signs of animal discomfort were noted, i.e., sudden rise in heart rate (HR), respiratory rate, or tail or paw movement. The animals’ chests and backs were thoroughly shaved to allow for direct contact of the defibrillator electrodes used for defibrillation during CPR.

After placing the rats on a surgical board in the supine position, the trachea was orally intubated using a modified 14 G cannula (Abbocath-T, Abbott Hospital Division, North Chicago, IL, USA). Animals were mechanically ventilated with a specialized ventilator capable of precise delivery of NO (Servo Ventilator 300A, Siemens, Munich, Germany) with an inspired oxygen fraction (FiO_2_) of 0.3. Respiratory frequency was adjusted to maintain end-tidal PCO_2_ between 35 and 40 mmHg, continuously monitored using an infrared CO_2_ analyzer (Cap Star 100, CWE Inc., Ardmore, PA, USA). A three-lead electrocardiogram was continuously measured using monopolar needle electrodes (MLA1204 Needle Electrodes, AD Instuments, Oxford, UK). The left femoral artery was surgically exposed, cannulated with a polyethylene catheter (PE 50) and connected to a high sensitivity transducer (Capto SP 844 Physiologic Pressure Transducer, Capto Inc., Skoppum, Norway) for the measurement of mean and diastolic arterial pressure (MAP and DAP). The left femoral vein was cannulated with an additional PE 50 catheter to allow for administration of fluids and epinephrine during CPR. Rectal temperature was monitored and maintained between 37.0 and 37.5 °C with the aid of a heating mat (TCAT-2LV-controller, Physitemp Science Products, Hofheim, Germany). All catheters were flushed intermittently with saline solution containing 2.5 IU · ml^−1^ of heparin.

### Experimental procedure

After preparation, animals were randomly assigned to three groups using the sealed envelope method. Animals received either 20 ppm (n = 10) or 40 ppm (n = 10) iNO, started with initiation of ventilation during CPR or no iNO treatment (control, n = 10).

Ventricular fibrillation (VF) was induced by transesophageal electrical stimulation. After placing the electrode using fluoroscopy guidance, alternating current (10 V, 50 Hz) was delivered to the heart using a commercially available fibrillator (Fi 20 M, Stockert GmbH, Freiburg, Germany). CA was confirmed by an abrupt decrease in MAP to less than 20 mmHg. Simultaneously, ventilation was stopped. After 10 minutes of untreated CA, CPR was initiated including mechanical ventilation with an FiO_2_ of 1.0 at a respiratory rate of 50 · min^−1^, and chest compressions delivered by a custom made mechanical thumper at a stroke rate of 200 · min^−1^. Animals in the iNO-treated groups were additionally given either 20 ppm or 40 ppm NO until 30 minutes after ROSC. All animals received an intravenous bolus of 0.02 mg · kg^−1^ epinephrine administered via the femoral access 30 seconds after starting chest compressions, which was repeated when MAP fell below 50 mmHg. After 3 minutes of CPR, external defibrillation with 5 J (Zoll MSeries, Zoll Medical Corporation, Chelmsford, MA, USA) was attempted up to three times. If ROSC was not achieved, administration of epinephrine at the same dosage and chest compressions were repeated for 1 minute before additional direct current counter shocks (again up to three times) were delivered. This cycle was repeated up to three times. ROSC was confirmed by spontaneous cardiac rhythm in conjunction with a rise in MAP to greater than 50 mmHg. If no ROSC was achieved within 3 minutes after the first shock, resuscitation attempts were stopped. Thirty minutes after successful resuscitation, FiO_2_ was reduced to 0.3 in all groups, and iNO administration was stopped. Animals were weaned from the ventilator and returned to their cages.

### Measurements

Ischemia time was calculated as the sum of the duration of VF (10 minutes), CPR (3 minutes) and the additional time needed to achieve ROSC (at most 3 additional minutes of CPR). Heart rate, MAP, DAP and end-tidal CO_2_ were continuously recorded on a multichannel recorder (Power Lab, AD Instruments, Spechbach, Germany). Arterial blood samples were drawn at baseline, 30 minutes, and 1 hour after ROSC. Partial pressure of arterial oxygen (P_a_O_2_) and of carbon dioxide (P_a_CO_2_), glucose, lactate levels and base excess were measured using a conventional blood gas analyzer (ABL700, Radiometer, Copenhagen, Denmark).

At the same time points and on day 7 post arrest, additional blood samples (0.5 mL) were drawn and centrifuged for 10 minutes at 2500 × G and 4 °C. The supernatant serum was immediately stored at −80 °C for subsequent analysis of tumor necrosis factor-alpha (TNF-α), interleukin (IL)-1ß and IL-6 using a magnetic bead-based multiplex assay (PCYTMAG-3 K, Merck-Millipore, Darmstadt, Germany) and a flow cytometry-based analyzer (LUMINEX® 100/200TM system, Austin, TX, USA). Migration inhibitory factor (MIF) levels in the serum samples were assessed using a modified mouse/human combination ELISA technique as described previously [21].

### Neurological testing

#### Neurological deficit score

On the 7 days following CPR, neurological performance was evaluated daily by investigators blinded to the animal’s treatment using a neurological deficit score (NDS) previously established in an asphyxial CA model [22] and validated by our group [23]. The test consists of six items rep, and coordination. Each item is graded depending on the severity and given a score. The score ranges from 0 (worst neurological impairment) to 500 (no neurological impairment). Deceased animals did not receive a score and were excluded from further analysis.

### Histopathology

Seven days after successful resuscitation, rats were re-anesthetized as described above. A midline thoracotomy was performed and the animals were transcardially perfused with 100 mL of NaCl 0.9 %.

#### Neurohistopathology

After transcardiac perfusion, brains were carefully removed and trans sagitally cut in half. Right hemispheres were post fixed in buffered 4 % paraformaldehyde. Standardized coronal slices were taken at a thickness of 2 mm resulting in a total of 8 slices per brain. The neocortex, hippocampal CA1 and anterior and posterior CA 3/4 sectors, and basal ganglia were chosen as regions of interest, and analyzed by an experienced neuropathologist blinded to the animal’s treatment assignment. Conventional hematoxylin/eosin (HE) and NeuN (Mouse anti-Neuronal Nuclei, monoclonal, Cat # MAB 377, Millipore, Darmstadt, Germany) staining was performed. A neuronal damage index was semiquantitatively assessed as previously established in this model [20, 23]. The proportion of neuronal cells with shrunken and/or hypereosinophilic cytoplasm (HE staining) in combination with a loss of NeuN-immunoreactivity was determined and summarized in a score: 0–5 % = 1; 5–10 % = 2; 10–20 % = 3; 20–30 % = 4; 30–40 % = 5; 40–50 % = 6; 50–60 % = 7; 60–70 % = 8; 70–80 % = 9; 80–90 % = 10; and 90–100 % = 11. Additionally activation of caspase 3 was assessed by immunohistochemistry using a rabbit monoclonal antibody against cleaved caspase 3 (Rabbit anti-cleaved Caspase-3 (Asp175), monoclonal, Cat # 9664, Cell Signaling Technology, Danvers, MA, USA) according to the protocol recommended by the manufacturer. Brain slices from an animal 6 hours after induction of subarachnoid hemorrhage served as positive control for the staining [24]. Animals not surviving until day 7 were excluded from histopathological analysis.

#### Myocardial histopathology

After transcardial perfusion, the hearts were gently removed and transversally bisected. Both atria and remnants of the aortic arch were removed, before fixation in 4 % buffered formalin for at least 24 hours. After paraffin embedding, standardized cross-sections of the ventricular parts were taken at a thickness of 2 μm resulting in a total of 10 slices per heart. Luxol fast blue (LFB) staining and counterstaining with nuclear fast red was performed according to a standard protocol [25]. Planimetric analysis was performed by two blinded investigators, and validated by an experienced pathologist, using ImageJ software (version 1.46r, National Institutes of Health, Bethesda, MD, USA). LFB positive controls (infarcted rat hearts) were used to calibrate the software to a threshold value for the detection of blue color (blue transverse banding and diffuse blue myocytes) to distinguish myofibrillar degeneration (LFB, blue), and normal tissue (nuclear fast red, purple). The values for the planimetric analysis of the two experimenters were averaged and tissue damage was expressed as percentage of blue staining from the total area.

### Statistical analysis

All data are expressed as mean ± standard error of the mean (SEM). Continuous data were analyzed using unpaired two-way repeated measures analysis of variance (ANOVA) with the Tukey post hoc test. Parameters at distinct time points were tested for significant differences between the groups by two-way ANOVA with the Tukey correction. Differences in survival rates were analyzed by the log-rank test followed by post hoc analysis with the log rank Bonferroni correction. Statistical analyses were performed using IBM SPSS statistic 20 and GraphPad Prism 6.04 (GraphPad Software Inc.) with a two-tailed hypothesis. In all cases, a *p* value ≤0.05 was considered to indicate statistical significance. On the basis of data derived from pilot experiments, power and sample size calculations were performed using PS: Power and Sample Size Calculation version 2.1.31 software by Dupont and Plummer [26].

## Results

**Fig. 1:**
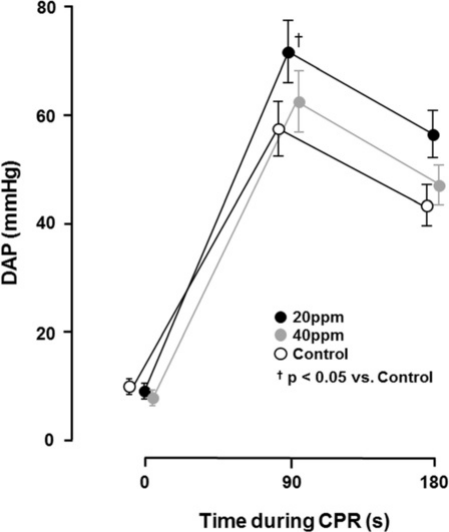
Diastolic arterial pressures (*DAP*) during cardiopulmonary resuscitation (*CPR*). Application of 20 ppm inhaled nitric oxide (iNO) during CPR significantly increased DAP in comparison to untreated controls; ^†^ *p* <0.05 for 20 ppm iNO vs. control; mean ± standard error of the mean. Data based on 10 animals in each group

**Fig. 2:**
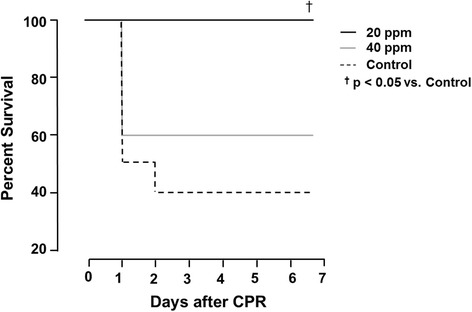
Seven-day survival in all groups. Animals treated with 20 ppm inhaled nitric oxide (iNO) had significantly better 7 day survival in comparison to control animals; ^†^ *p* <0.05 for 20 ppm iNO vs. control. *CPR* cardiopulmonary resuscitation

Application of 20 ppm iNO significantly increased DAP during CPR (Fig. 1) and significantly decreased time to ROSC in comparison to untreated controls (20 ppm: 792 ± 5 s vs. 40 ppm: 798 ± 9 s vs. control: 842 ± 21 s; *p* = 0.02 20 ppm vs. control). This translated into pronounced increases in ROSC rates (control: n = 7, 20 ppm: n = 10, 40 ppm n = 6) and significant differences in the 7-day survival in comparison to animals receiving no iNO treatment (Fig. 2).

There were no significant differences in the number of doses of epinephrine during CPR between groups of animals achieving ROSC (20 ppm: 1.14 ± 0.3 vs. 40 ppm: 1.75 ± 0.3 vs. control: 2.29 ± 0.5).

**Fig. 3:**
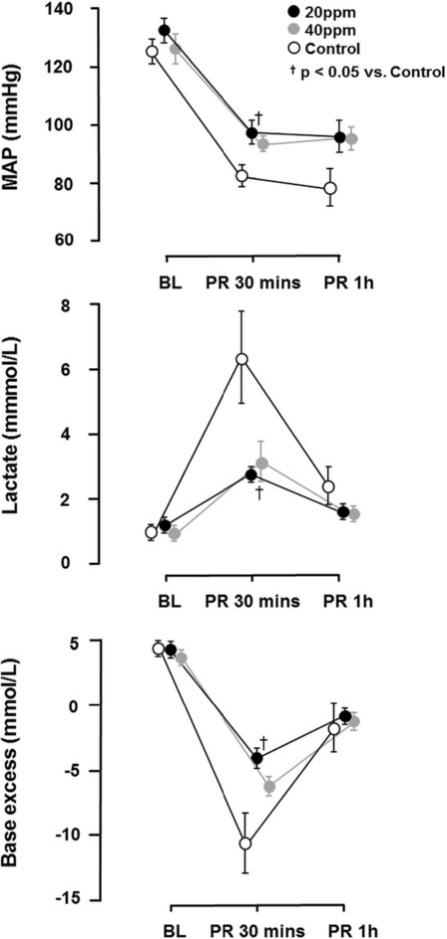
Alterations in mean atrial pressure (*MAP*), lactate levels and base excess before cardiac arrest and after return of spontaneous circulation (ROSC). The decrease in MAP and concurrent increase in lactate was significantly less pronounced in animals treated with 20 ppm inhaled nitric oxide (iNO) in comparison to controls. Inhalation of NO attenuated the decrease in base excess, being significant in animals that received 20 ppm iNO in comparison to controls. ^†^ *P* <0.05 for 20 ppm iNO vs. control; mean ± standard error of the mean. Data based on 10 animals in each group at baseline (*BL*). All other time points: 20 ppm, n = 10; 40 ppm, n = 6; Control, n = 7. PR post return of ROSC

No significant differences were observed in hemodynamics, variables of gas exchange, or glucose and lactate concentrations, between iNO-treated animals and the control group at baseline (Table 1, Fig. 3). In control animals, we observed a transient decrease in MAP with a dramatic increase in lactate and glucose levels following ROSC. While both iNO-treated groups presented with higher MAP, lower lactate levels and concurrently higher base excess post ROSC, we only observed a statistically significant difference for these parameters in 20-ppm-treated animals versus controls (Fig. 3). Furthermore, inhalation of NO attenuated the frequently observed increase in glucose levels after ROSC, however this was not statistically significant.

**Table 1:** Physiologic data

		BL	PR 30	PR 60
HR (bpm)	Control	378 ± 11	336 ± 9	364 ± 20
	20 pm iNO	360 ± 15	344 ± 12	372 ± 15
	40 ppm iNO	389 ± 9	383 ± 11	402 ± 12
Hgb (g/dL)	Control	14.5 ± 0.2	15.8 ± 0.4	16.2 ± 0.5
	20 pm iNO	14.6 ± 0.3	15.9 ± 0.2	15.5 ± 0.4
	40 ppm iNO	14.2 ± 0.3	15.3 ± 0.5	15.2 ± 0.4
CO-Hba (%)	Control	0.4 ± 0.1	0.1 ± 0.1	0.4 ± 0.1
	20 pm iNO	0.5 ± 0.1	0.8 ± 0.3	0.0 ± 1.1
	40 ppm iNO	0.5 ± 0.1	0.1 ± 0.5	0.4 ± 0.3
Glucose (mg/dL)	Control	139 ± 5	306 ± 28	190 ± 29
	20 pm iNO	129 ± 5	240 ± 14	179 ± 15
	40 ppm iNO	125 ± 7	204 ± 16	141 ± 13
HCO3ˉ	Control	27.4 ± 0.3	14.8 ± 1.7	22.2 ± 0.1
	20 pm iNO	27.1 ± 0.4	19.2 ± 1.0	23.0 ± 0.4
	40 ppm iNO	26.7 ± 0.6	18.7 ± 0.6	22.7 ± 0.1
Ph	Control	7.4 ± 0.0	7.2 ± 0.1	7.4 ± 0.0
	20 pm iNO	7.4 ± 0.1	7.3 ± 0.0	7.4 ± 0.0
	40 ppm iNO	7.4 ± 0.1	7.3 ± 0.0	7.4 ± 0.0
PaO_2_ (mmHg)	Control	131 ± 8	301 ± 36	121 ± 5
	20 pm iNO	136 ± 9	253 ± 38	122 ± 11
	40 ppm iNO	124 ± 6	271 ± 21	132 ± 10
PaCO_2_ (mmHg)	Control	42 ± 2	42 ± 3	41 ± 3
	20 pm iNO	42 ± 2	48 ± 3	40 ± 2
	40 ppm iNO	41 ± 1	40 ± 2	38 ± 1
Temperature (°C)	Control	37.4 ± 0.1	37.2 ± 0.2	37.4 ± 0.1
	20 pm iNO	37.3 ± 0.1	37.4 ± 0.1	37.4 ± 0.1
	40 ppm iNO	37.4 ± 0.0	37.3 ± 0.1	37.3 ± 0.1

Physiologic data for control and iNO-treated animals at baseline and after cardiopulmonary resuscitation. Temperature indicates body temperature estimated using a rectal probe. ^*^ *P* <0.05 vs. control; mean ± standard error of the mean. Data based on 10 animals in each group on BL. PR30 and PR60: control, n = 7; 20 ppm, n = 10; 40 ppm, n = 6. *BL* baseline, *PR* time post resuscitation in minutes, *HR* heart rate, *Hgb* hemoglobin, *CO-Hba* carboxyhemoglobin in arterial blood, *PaO*_*2*_ arterial oxygen tension, *PaCO*_*2*_ arterial carbon dioxide tension

**Fig. 4:**
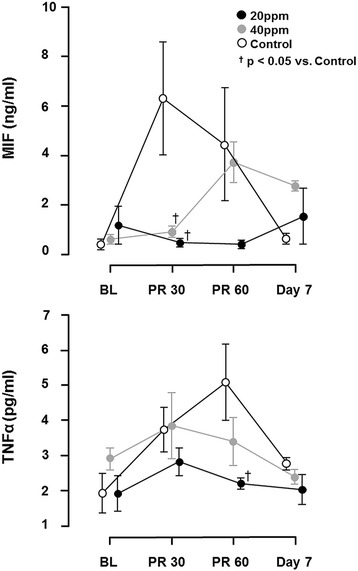
Release of cytokines, migration inhibitory factor (*MIF*) and TNF-α. Successfully resuscitated animals had rising values of circulating TNF-α and MIF, which was significantly diminished by inhalation of 20 ppm nitric oxide (NO). ^†^ *P* <0.05 for 20 ppm inhaled nitric oxide (iNO) vs. control; mean ± standard error of the mean. Data based on 10 animals in each group at baseline (*BL*). Thirty minutes (*PR30*) and 60 minutes (*PR60*) post return of spontaneous circulation: 20 ppm, n = 10; 40 ppm, n = 6; Control, n = 7. Day 7: 20 ppm, n = 10; 40 ppm, n = 6; Control, n = 4

Successfully resuscitated animals exhibited an increased release of MIF and TNF-α, which was reduced by inhalation of NO (Fig. 4). IL-1ß and IL-6 levels were below detection levels of the magnetic bead-based multiplex assay (IL1-ß 2,4 - 10.000 pg/mL; IL-6 73,2 - 30.000 pg/mL) at the given time points.

We observed severe neurological dysfunction as measured with the NDS in all control animals during the 7 days after CA and CPR. In contrast, 20 ppm iNO markedly improved neurological function 2–7 days after CA and CPR (Table 2).

**Table 2:** Neurologic deficit score

	Day 1	Day 2	Day 3	Day 4	Day 5	Day 6	Day 7
Control	300 ± 99	308 ± 104	373 ± 85	390 ± 94	390 ± 94	390 ± 94	403 ± 82
20 pm iNO	393 ± 157	473* ± 43	479* ± 37	482* ± 23	485* ± 24	487* ± 18	488* ± 17
40 ppm iNO	376 ± 85	398 ± 70	430 ± 54	454 ± 40	471 ± 36	463 ± 43	467 ± 45

Neurological deficit score on all days following cardiac arrest and cardiopulmonary resuscitation. Control animals exhibited severe neurological dysfunction. Inhaled nitric oxide (iNO)-treated animals had better neurological outcomes, with a statistically significant difference in animals treated with 20 ppm iNO on postoperative days 2–7; *p <0.05 for 20 ppm iNO vs. control; mean ± SD. Day 1: Control, n = 5; 20 ppm iNO, n = 10; 40 ppm iNO, n = 6. Days 2–7: Control, n = 4; 20 ppm iNO, n = 10; 40 ppm iNO, n = 6

**Fig. 5:**
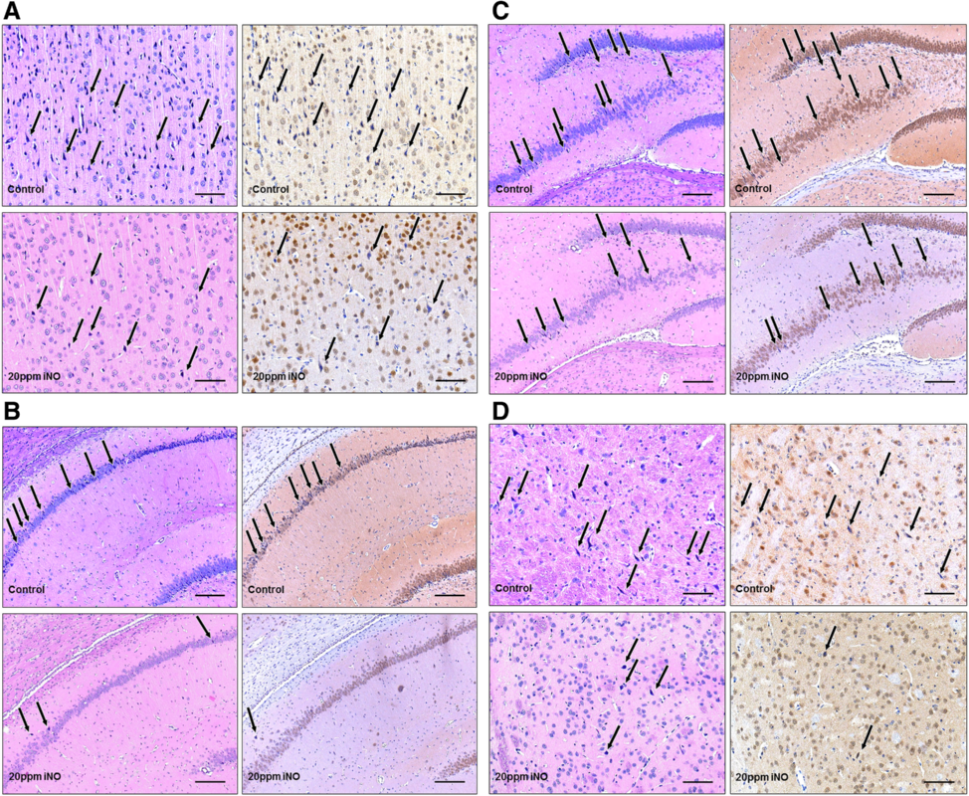
Representative photomicrograph of HE and NeuN staining of the neocortex (**a**), hippocampal CA1 (**b**) and anterior and posterior CA 3/4 sectors (**c**), and basal ganglia (**d**) showing numerous damaged neurons (*black arrows*) in a control animal and less necrosis in an animal treated with 20 ppm inhaled nitric oxide (*iNO*). *Scale bars* 90 μm (**a**), 180 μm (**b**), 180 μm (**c**), 90 μm (**d**). Cerebral tissue harvested 7 days post cardiac arrest

**Fig. 6:**
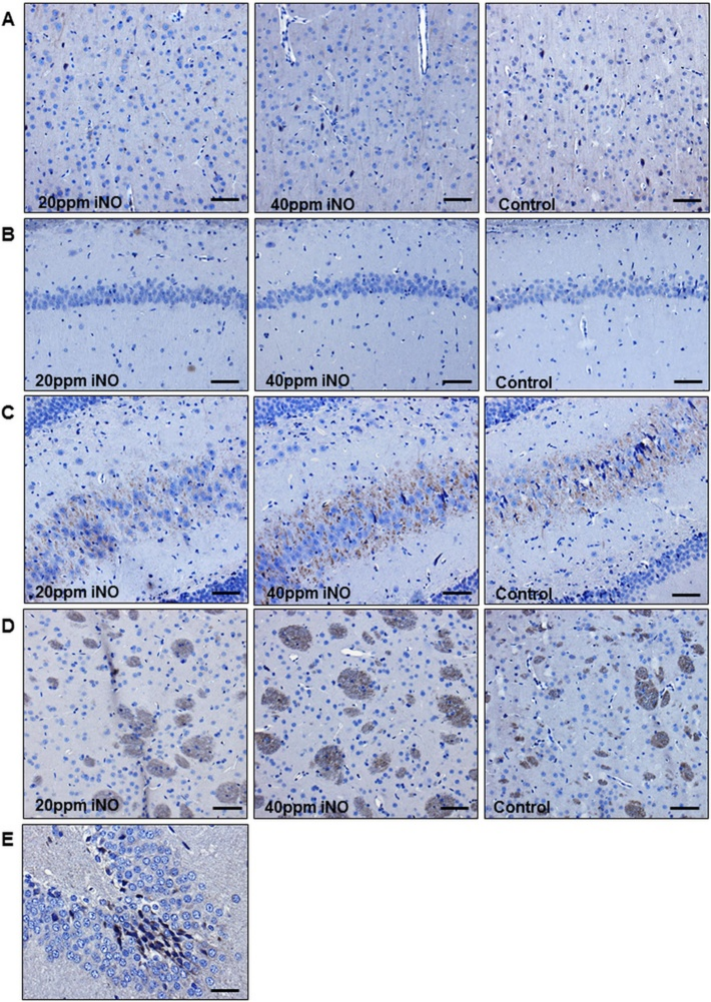
Representative photomicrograph of immunohistochemical staining for cleaved caspase 3 of the neocortex (**a**), hippocampal CA1 (**b**) and anterior and posterior CA 3/4 sectors (**c**), and basal ganglia (**d**). No caspase activation was found in any of the groups. *Scale bar* 90 μm. **e** Gyrus dentatus of an animal 6 hours after induction of subarachnoid hemorrhage as positive control for the staining. *Scale bar* 30 μm. Cerebral tissue harvested 7 days post cardiac arrest. *iNO* inhaled nitric oxide

Neurohistopathological evaluation revealed that 40 ppm iNO attenuated the neocortical injury in surviving rats 7 days after CA and CPR (Table 3, Fig. 5a-d). There was no difference between treatment groups in the extent of histological brain injury in other regions of the brain. We did not observe caspase-3 activation in brain sections from any of the groups 7 days after CA (Fig. 6).

**Table 3:** Neuronal damage index

NDI	Neocortex	CA 1	CA 3/4	Basal ganglia
Control	2.25 ± 0.25	2.00 ± 0.71	2.50 ± 1.10	1.00 ± 0.00
20 pm iNO	2.00 ± 0.15	2.50 ± 0.50	1.60 ± 0.31	1.10 ± 0.10
40 ppm iNO	1.60* ± 0.25	3.20 ± 1.20	1.60 ± 0.40	1.00 ± 0.00

Conventional hematoxylin/eosin and NeuN (Mouse anti-Neuronal Nuclei) staining was performed. A neuronal damage index (*NDI*) was semiquantitatively assessed in neocortex, hippocampal CA1 and CA 3/4 sectors. *P <0.05 for 40 ppm inhaled nitric oxide (*iNO*) vs. control mean ± standard error of the mean. Data based on cerebral tissue taken from Control animals (n = 4), and animals treated with 20 ppm (n = 10), and 40 ppm (n = 5) iNO

**Fig. 7:**
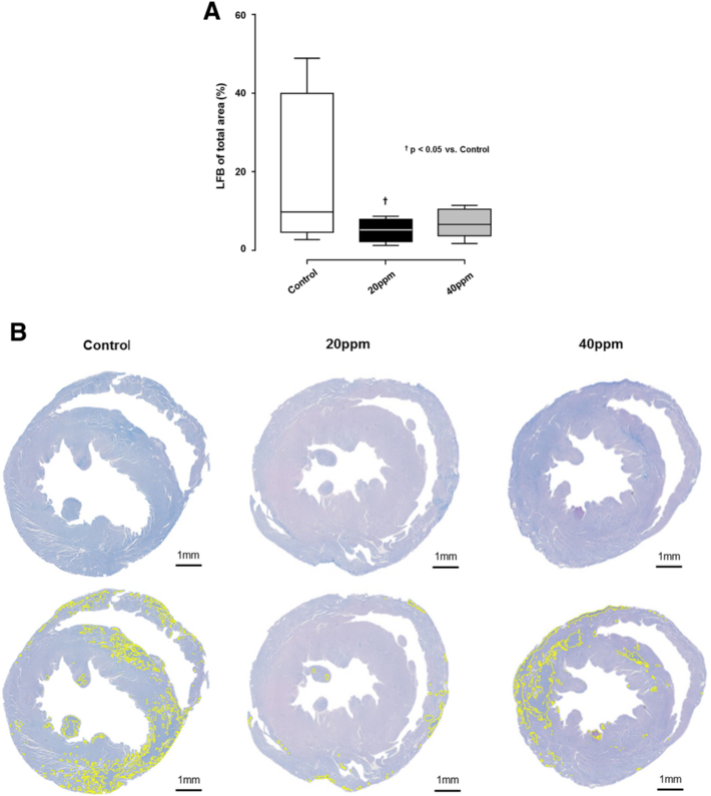
Successful cardiopulmonary resuscitation resulted in cardiac myofibrillar degeneration detected by luxol fast blue staining (*LFB*). Cardiac tissue harvested 7 days post cardiac arrest. **a** Cardiac damage was significantly less pronounced in animals treated with 20 ppm inhaled nitric oxide (iNO) in comparison with untreated controls. *Bo*x: *bottom* and *top* represent the first and third quartiles, *band* represents the median, *whiskers* mark the minimum and maximum of the data. ^†^ *P* <0.05 for 20 ppm iNO vs control. Control, n = 4; 20 ppm, n = 10; 40 ppm, n = 6. **b** Representative photomicrographs of heart cross-sections with LFB and hematoxylin/eosin (HE) counterstaining of a control, and from a 20-ppm, or 40-ppm iNO-treated animal. The planimetry analysis with imageJ software discriminates between myofibrillar degeneration (LFB blue staining, *yellow markers*) and undamaged tissue (HE counterstaining, *purple*)

We observed that CA and CPR caused myocardial damage in control rats as demonstrated by LFB staining which was significantly attenuated by inhalation of 20 ppm NO (Fig. 7a and b).

## Discussion

Our study demonstrates that ventilation with 20 ppm iNO, but not 40 ppm iNO during resuscitation until 30 minutes after ROSC, increases DAP during CPR, resulting in a decreased time to ROSC. The beneficial effects of iNO were associated with higher MAPs, lower lactate levels, and a reduced inflammatory response post ROSC in comparison to control animals. Furthermore, CA-induced cardiac damage was markedly attenuated in animals treated with 20 ppm iNO and brain neocortical injury was ameliorated by 40 ppm iNO. Last, breathing 20 ppm NO improved neurological outcomes and 7-day survival rates in rats.

Our study corroborates previous findings of beneficial effects of iNO on neurological damage and mortality when given after CA in mice [27]. Minamishima and colleagues exposed spontaneously breathing mice for 23 hours to 40 ppm NO starting 1 hour after CPR. They observed marked increases in brain water diffusion in the hippocampus, caudoputamen and cortex, indicative of tissue edema in control animals after CA and CPR. Breathing NO markedly ameliorated brain injury and improved neurological function and 10-day survival rate in mice [18]. The same group recently reported that combining iNO with MTH improved outcomes after CA in mice compared to MTH alone [27]. The current study sought to explore the immediate and delayed effects of iNO given during and after CA. While administration of iNO during CPR may currently be difficult to realize in a clinical setting, in the near future a recently developed novel NO-delivery device may enable administration of iNO in the field, without the need for cumbersome gas tanks [28].

Several other investigations report beneficial effects of iNO on neurological outcomes in parallel with histopathologic alterations in models of stroke or traumatic brain injury [29–31]. Our study confirms these protective effects of iNO on outcome in the setting of VF-induced CA and CPR in rats, a model that is arguably more clinically relevant than KCL-induced arrest in mice. In our study, the beneficial effects of iNO on the neurological outcome was only partially reflected in the histopathological findings; neocortical injury was ameliorated by iNO at 40 ppm, but not at 20 ppm. This might be due to the fact that several animals in both the iNO and the control group died during the 7-day observation period, and were therefore unavailable for further histopathologic examination (Fig. 2). It is likely that we did not observe cleaved caspase-3 in the brain sections 7 days after CA because caspase-3 is activated at earlier time points after ischemic insult [18] (Fig. 6).

The beneficial effects of NO on the brain are likely mediated by several mechanisms. In animal models of stroke in mice and sheep, iNO administration led to significant improvements in both regional cerebral blood flow [31] and microvascular blood flow [29] in the penumbra via cyclic guanosine monophosphate-dependent cerebral vasorelaxation. Alternatively, iNO may attenuate inflammatory response [32]. Recent studies suggested salutary effects of iNO on markers of inflammation and oxidative stress in patients undergoing cardiac surgery [33]. In line with this, our study reveals that 20 ppm iNO significantly attenuates the release of TNF-α and MIF in rats after CA and CPR. MIF is an upstream regulator of the initial immune response with various pro-inflammatory effects [34], which initiates and exacerbates chronic and acute inflammatory disorders [35]. MIF regulates the production and release of TNF-α, IL-1β, IL-2, IL-6, IL-8, IL-12 and interferon (IFN)-γ [34, 35]. Furthermore MIF has unique characteristics in its rapid release profile in response to pathogenic stimuli (e.g., hypoxia, infection, or inflammation) from several cell types including T cells, macrophages, endothelial cells, thrombocytes and cardiomyocytes, which is due to its storage in preformed intracellular pools [34, 35]. In the heart, MIF is released by ischemic cardiomyocytes and acts by autocrine/paracrine signaling. Inhibition of MIF pro-inflammatory activities after ischemia has been shown to improve cardiomyocyte survival and myocardial function [36, 37]. Therefore, it is suggested that elevated post-ischemic MIF levels exacerbate ischemic tissue damage after myocardial ischemia and reperfusion. [38] Along these lines, our observations suggest that iNO may exert cardioprotective effects after CA and CPR by inhibiting MIF activity. Additionally, iNO may preserve the blood-brain barrier by preventing apoptosis of cerebral tissue, a mechanism that has been previously described as a driver of cognitive dysfunction after subarachnoid hemorrhage [39].

The protective effects of iNO in our study may also be explained by improved hemodynamics during and after resuscitation. We noted that iNO significantly improved DAP during CPR and this might be a result of increased left ventricular filling. iNO is known to lower pulmonary artery pressure, hence enhancing transpulmonary blood flow. Indeed, dramatic increases in pulmonary artery pressure with concurrent reductions in pulmonary blood flow are well-documented during closed chest compressions in humans and animals [40–42]. Therefore it is conceivable that iNO-induced pulmonary vasodilation improved hemodynamics in our study by enhancing left ventricular filling leading to the improved ROSC rates. In fact, in a recent study in a large animal model of CA and CPR, we observed that iNO markedly decreased pulmonary vascular resistance and increased coronary perfusion pressure [43]. Furthermore, the pulmonary vasodilating effects of iNO might also account for improved hemodynamics and metabolic alterations until 30 minutes post arrest, as we observed significantly higher MAPs, while the arterial lactate level was reduced by almost 100 %. Here again, reductions in right ventricular afterload might explain these findings, as right ventricular dysfunction is common in survivors of CA [44].

Inhaled nitric oxide may improve cardiac function via extrapulmonary effects. For instance, iNO has been shown to improve cardiac function not only through unloading the right ventricle (RV) and thus improving left ventricular (LV) preload, but also through direct effects on contractility [45]. Consistent with a previous study by Dezfulian and colleagues, our results showed that iNO markedly attenuated myocardial injury as demonstrated by LFB staining. By its ability to act as an anti-oxidant, iNO may also prevent injury to vascular smooth muscle cells caused by reactive oxygen species [46], and may therefore preserve an adequate vascular tone. Tissue perfusion may furthermore be improved by the ability of iNO to inhibit platelet aggregation [47].

We propose a dual mechanism of action for iNO in the setting of CA and CPR, which first allows for improved resuscitation success by increased left ventricular filling and then exerting secondary actions on cerebral microvasculature and the inflammatory response. While both effects appear to contribute to the pro-survival effects of iNO, the hemodynamic effects appear to be more crucial in this particular setting, as optimal hemodynamics during CPR are the prerequisite for ROSC. Additionally, altered tissue perfusion can modify reactions on cellular and sub-cellular levels as a secondary effect. How iNO exerts extrapulmonary effects remains incompletely understood. It has been suggested that NO is transported to remote organs as more stable bioactive molecules including iron nitrosyl [48], S-nitrosothiols [49], nitrite [50], and nitrosolipids [51]. Previous studies showed that beneficial effects of iNO after CA and CPR were associated with marked increase in serum nitrite levels [18, 27]. Although the exact mechanisms of endocrine effects of NO remain elusive, the current study supports the hypothesis that iNO exerts extra-pulmonary effects in remote organs after CA and CPR.

In the current study, the most prominent pro-survival effects were observed with 20 ppm iNO, while histological neocortical injury was ameliorated by 40 ppm iNO. This is in contrast to previously published data from Kida and colleagues who found optimal survival rates after CA in mice using 40 and 60 ppm iNO concomitantly with MTH [27]. These differences might be explained by the different timing of NO inhalation, use of MTH in the Kida study, or limited sample size in both studies. In general, positive effects were observed across a wide range of iNO concentrations (5–50 ppm) in all of the abovementioned investigations [18, 27, 29–31, 52, 53]. The reason why 40 ppm iNO did not improve survival in the current study is unknown at this point. However, it is important to note that iNO at 40 ppm did not worsen survival after CA compared to the air-breathing controls, suggesting that iNO at 40 ppm is not exerting any toxic effects. While breathing very high concentrations of NO (≥80 ppm) at the time of reperfusion may exert toxic effects, presumably by increasing peroxinitrite [31], it has never been observed at 40 ppm iNO in a large number of preclinical and clinical studies [54].

We recognize several limitations in the interpretation of our findings. First, data were obtained from healthy animals, and translation of results to humans with pre-existing diseases has always to be performed with caution. Second, it might well have been that iNO also influenced plasma cytokine levels of IL-1ß and IL6, which we did not detect in our short time frame until 1 hour post CA. Third, post-resuscitation care was not provided in our protocol, possibly resulting in premature death of animals in the control and 40-ppm iNO groups. Having additional survivors on day 7 might have very well influenced the results. However, early neurologic failure is the most common mode of death in humans [55]. As CA was electrically induced in this model, deaths in the early post-resuscitation phases may be the result of cardiovascular collapse rather than pure neurocognitive dysfunction [56]. However, as deceased animals were excluded from further neurocognitive evaluation, this uncertainty did not affect the results of the NDS-based neurocognitive evaluation. As stated above, our study cannot discern whether the effects on hemodynamic function and neurologic outcomes are solely attributable to direct effects of iNO at a cellular level or whether improved myocardial performance resulted in better organ perfusion, which in turn resulted in improved functional recovery. We believe both alterations act in concert to achieve the benefits of iNO during CPR seen in this investigation. Although central venous pressure measurements may provide further information, we chose to minimize trauma to optimize our model for the examination of the effects of iNO on long-term neurological outcomes.

## Conclusions

We conclude that 20 ppm iNO administered at the onset of CPR and continued for 30 minutes after successful resuscitation led to increased CPR success and improved 7-day survival in rats. In addition, the inflammatory response and cardiac damage were attenuated, and neurological performance was significantly improved by NO inhalation. Although 40 ppm iNO attenuated histological neocortical injury after CA, it did not improve survival rates and neurological function compared to air. It is important to note that 20 ppm is the standard starting concentration of iNO in vast majority of clinical settings. Thus, our findings that breathing NO at 20 ppm for a short period of time during CPR markedly improves long-term outcomes after CA is highly clinically relevant. These findings should encourage clinical evaluation of iNO, given the well-known and established safety profile of NO inhalation in adult and pediatric patients. Additional large-animal studies are also warranted to elucidate the mechanisms responsible for the beneficial effects of inhaled NO.

## Key messages

- 20 ppm of iNO increases CPR success and survival
- 20 ppm of iNO improves clinical outcomes after CPR in rats
- Given the well-known and established safety profile of NO inhalation in adult and pediatric patients, these findings should encourage clinical evaluation

## Abbreviations

ANOVA: analysis of variance
CA: cardiac arrest
CPR: cardiopulmonary resuscitation
DAP: diastolic arterial pressure
ELISA: enzyme-linked immunosorbent assay
FiO_2_: inspired oxygen fraction
HE: hematoxylin/eosin
HR: heart rate
IFN: interferon
IL: interleukin
iNO: inhaled nitric oxide
LFB: Luxol fast blue
MAP: mean arterial pressure
MTH: mild therapeutic hypothermia
MIF: migration inhibitory factor
NDS: neurological deficit score
NDI: neuronal damage index
TNF-α: tumor necrosis factor-alpha
PaCO_2_: arterial carbon dioxide tension
P_a_O_2_: arterial oxygen tension
ROSC: return of spontaneous circulation
SEM: standard error of the mean
VF: ventricular fibrillation
